# Bridging psychometrics and language: a method for extracting leadership insights from open-text responses

**DOI:** 10.3389/fpsyg.2026.1730831

**Published:** 2026-05-15

**Authors:** Lauri Ahonen, Hannele Niemi, Vesa Nissinen

**Affiliations:** 1 Faculty of Educational Sciences, University of Helsinki, Helsinki, Finland; 2 Center of Avalanche Research and Education, UiT The Arctic University of Norway, Tromsø, Norway; 3 Deep Lead Inc, Helsinki, Finland

**Keywords:** psychometrics, natural language processing, construct validity, text embeddings, semantic similarity, sentiment analysis, leadership assessment

## Abstract

We present a compact, reproducible NLP method that turns open-text leadership feedback into theory-aligned signals and validates them against questionnaire scores. Inputs are multilingual 360° feedback. After preprocessing and translation, we (i) classify sentiment and (ii) compute construct salience scores by calculating cosine similarity between embedding space open-text feedback and seed-phrase representations of Deep Leadership Model (DLM) constructs. We test the estimated scores and classes against validated questionnaire results using three criteria: (1) association between sentiment and overall questionnaire outcomes with controls for open-text feedback type ; (2) construct salience score correlation with matching questionnaire factor scores versus non-matching and permutation baselines; and (3) interpretability via 360° role-wise construct profiles that align with established patterns. Results show that framework-aware open-text scoring complements existing DLM metrics and provide transparent, auditable diagnostics at the construct level. Because the approach relies on seedable constructs and questionnaire anchors, it generalizes beyond DLM: the same pipeline can augment any psychometric tool that pairs open-text responses with theory-defined dimensions, supporting scalable development, monitoring, and evidence-based use.

## 1 Introduction

Recent Natural Language Processing (NLP) methods make it feasible to convert narrative feedback into quantitative signals, but it remains unclear when such text-derived measures align with established psychometric constructs. We present a reproducible pipeline that extracts text-derived signals and validates them against scores from a theory-grounded questionnaire. We demonstrate the approach in leadership assessment using the Deep Leadership Model (DLM) and its questionnaire (DLQ) as a test case, asking whether text-derived signals covary with overall leadership quality and show convergent validity with the matching DLQ constructs.

Leadership research commonly distinguishes transactional and transformational approaches (Burns, 1978; Bass, 1985). Transactional leadership emphasizes contingent reinforcement and corrective monitoring, whereas transformational leadership emphasizes vision, intellectual stimulation, and individualized consideration (Burns, 1978; Bass, 1985). Both perspectives remain in use in contemporary leadership research (Khairy et al., 2023; Mekonnen and Bayissa, 2023; Ran et al., 2024).

In leadership research and training, the 360° feedback system is widely used to evaluate leaders' behavior, competencies, and effectiveness, with assessments drawn from multiple perspectives—often from supervisors, peers, subordinates, and self-assessments – to provide a holistic view of leadership behaviors and developmental needs (Bracken et al., 2016; Judge and Piccolo, 2004; London and Smither, 2002). Feedback is often collected via surveys that include core constructs of leadership theory and use open-text expressions. The 360° feedback system is also used with transactional and transformational leadership theories.

The Deep Leadership Model (DLM) is a leadership development framework grounded in transformational leadership theory and operationalized through the Deep Leadership Questionnaire (DLQ) (Bass, 1985; Bass and Avolio, 1990; Nissinen, 2001, 2006). DLM implementations typically collect multi-rater (360°) evaluations in which respondents provide both DLQ ratings and open-text comments (strengths, development areas, and organizational improvements). The DLQ yields factor scores for Professional Skills, Building Trust, Inspirational Motivation, Intellectual Stimulation, Individualized Consideration, Controlling Leadership, Passive Leadership, Satisfaction, Effectiveness, and Extra Effort. For our purposes, DLM/DLQ provides a clear set of theory-linked constructs with established questionnaire scoring and a parallel stream of narrative feedback authored in applied development contexts.

In parallel, organizational research has increasingly used NLP to quantify attitudes and performance-relevant perceptions from workplace text (Bhatia et al., 2022; Pang and Lee, 2008), and semantic approaches suggest that meaning in text can partially reproduce patterns observed in organizational behavior measures (Kobayashi et al., 2018). Recent work further demonstrates that narrative responses can be transformed into quantitative indicators of psychological constructs and job-related attitudes, and that deep models can scale aspects of assessment beyond traditional surveys (Speer, 2020; Speer et al., 2023; Abdurahman et al., 2024). Related findings in well-being and mental health show that distributional-semantic representations of brief open-text descriptions can converge meaningfully with established psychometric constructs (Kjell et al., 2019, 2021), and applied studies in education and entrepreneurship similarly illustrate NLP's capacity to surface implicit perceptions in textual data (Zhong et al., 2024; Luncheon and Kasztelnik, 2021). Accordingly, our goal is not to replicate DLQ scores from text, but to derive theory-anchored, interpretable text signals that can be compared with DLQ outcomes to support construct-level diagnostics.

Prior work integrating narrative feedback with survey instruments typically reports complementary rather than strongly convergent insights: open-text responses often surface emphases, framing, or concerns that are not fully captured by fixed questionnaire items, even when both are grounded in the same theoretical model. Our aim is therefore not to replace or replicate questionnaire scores, but to provide theory-aligned, interpretable text-derived signals that can be meaningfully compared with, and interpreted alongside, established psychometric measures.

More recently, leadership assessment has also begun to leverage AI by evaluating how participants lead AI-agent teams and relating performance to leadership effectiveness, or by classifying leadership styles from structured survey data (Weidmann et al., 2025; Alomairi and Ibrahim, 2025). Together, these strands motivate scalable approaches to psychometrical measurements. However, these applications rarely align open-text feedback directly to framework anchored factors and validate text-derived signals against the framework's questionnaire scores at the construct level.

We address this integration gap by deriving framework-anchored open-text signals that can be used alongside DLQ scores. Specifically, we represent each DLM construct using theory-informed seed-phrases and compute a construct salience profile for each open-text response as cosine similarity in embedding space. In addition, we estimate the evaluative tone (sentiment/valence) of each response. These two outputs (valence and construct salience) can be aggregated by prompt type, cohort, and rater role, and compared directly against DLQ factor scores and the General Leadership Index (GLI).

In this approach, sentiment analysis captures evaluative tone, while modern embedding methods operationalize semantic proximity between text and theory-defined constructs (Liu, 2022). Because leadership evaluations are partly affective and relational, we expect the valence of narrative feedback to covary with overall leadership quality (Breevaart et al., 2014), especially the feedback that is evaluative by nature. In contrast, prompts that solicit non-loaded suggestions may elicit a more instrumental, problem-solving language, weakening this association to overall leadership quality even when the semantic content remains informative.

Our goal is validation-oriented and we do not assume that narrative feedback is a substitute for questionnaire measurement. Instead, we test whether text-derived signals are (a) systematically related to established questionnaire metrics when theory suggests they should be, and (b) strongest for matching constructs relative to non-matching and permuted baselines. Accordingly, we evaluate the following validation targets (Research Questions):

- **RQ1 (Valence validity)**. Is the association between sentiment and GLI contingent on prompt type, such that (i) leader-evaluative prompts yield sentiment signals that positively correlate with GLI, whereas (ii) organization-focused prompts yield weaker or no association?
- **RQ2 (Construct convergent validity)**. Does the text-derived construct salience profile show convergent validity (i.e. correlations) with the Imatching DLQ factors?
- **RQ3 (Complementary interpretability)**. Do text-derived metrics provide interpretable complementary information beyond DLQ scores?

## 2 Method

We model open-text feedback accompanying the Deep Leadership Questionnaire (DLQ) to derive theory-aligned signals and test them against DLQ scores from multiple Deep Lead Ltd. training programs. Open-text fields include three prompts (strengths, development, and organization); numeric measures include DLQ factor scores and overall indices. The factor dimensions represent the following constructs: Professional Skills; Building Trust and Confidence; Inspirational Motivation; Intellectual Stimulation; Individualized Consideration; Corrective and Controlling Leadership; Passive Leadership; Satisfaction; Effectiveness and Extra Effort. For specific item wording, refer to the DLQ instrument in Nissinen (2001).

**Figure 1:**
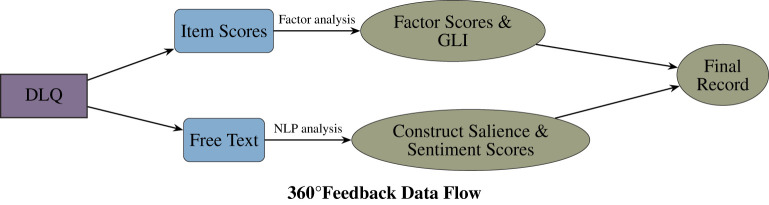
360° Feedback analytical pipeline. Visual reference of the transformation logic for a single rater-target record. Quantitative Item Scores undergo factor analysis to derive the General Leadership Index (GLI) and constituent factors. Outside traditional DLM pipeline, the three Free Text fields (Istrengths, Idevelopment opportunities, and Iorganizational improvements) are processed via NLP to extract Construct Salience and Sentiment Scores for the Final Record. Additionally Final Record contains covariates (e.g., 360° rater role and the cohort).

The data processing architecture is illustrated in Figure 1. Each DLQ record represents a single rater evaluating one target leader within a cohort. Open-text responses were analyzed at the record and prompt level rather than aggregated into leader-level “bags of text,” reflecting typical 360° feedback practice where comments are brief and role-specific. To mitigate effects of variations in verbosity by responders, construct salience scores were normalized within response (see Equation 3b for details).

Across all analyses, the primary observational unit is a record–prompt response, i.e., one open-text entry (strengths, development, or organization) written by one rater within a single DLQ record, paired with the same record's DLQ factor scores and GLI. Thus, a single DLQ record can contribute up to three observations (one per prompt) when multiple prompts are completed, and contributes fewer observations when some prompt responses are missing. We do not aggregate texts; all NLP quantities (sentiment and construct-salience profiles) are computed at the individual response level and only aggregated for descriptive plots (e.g., by rater role or the cohort).

**Figure 2:**
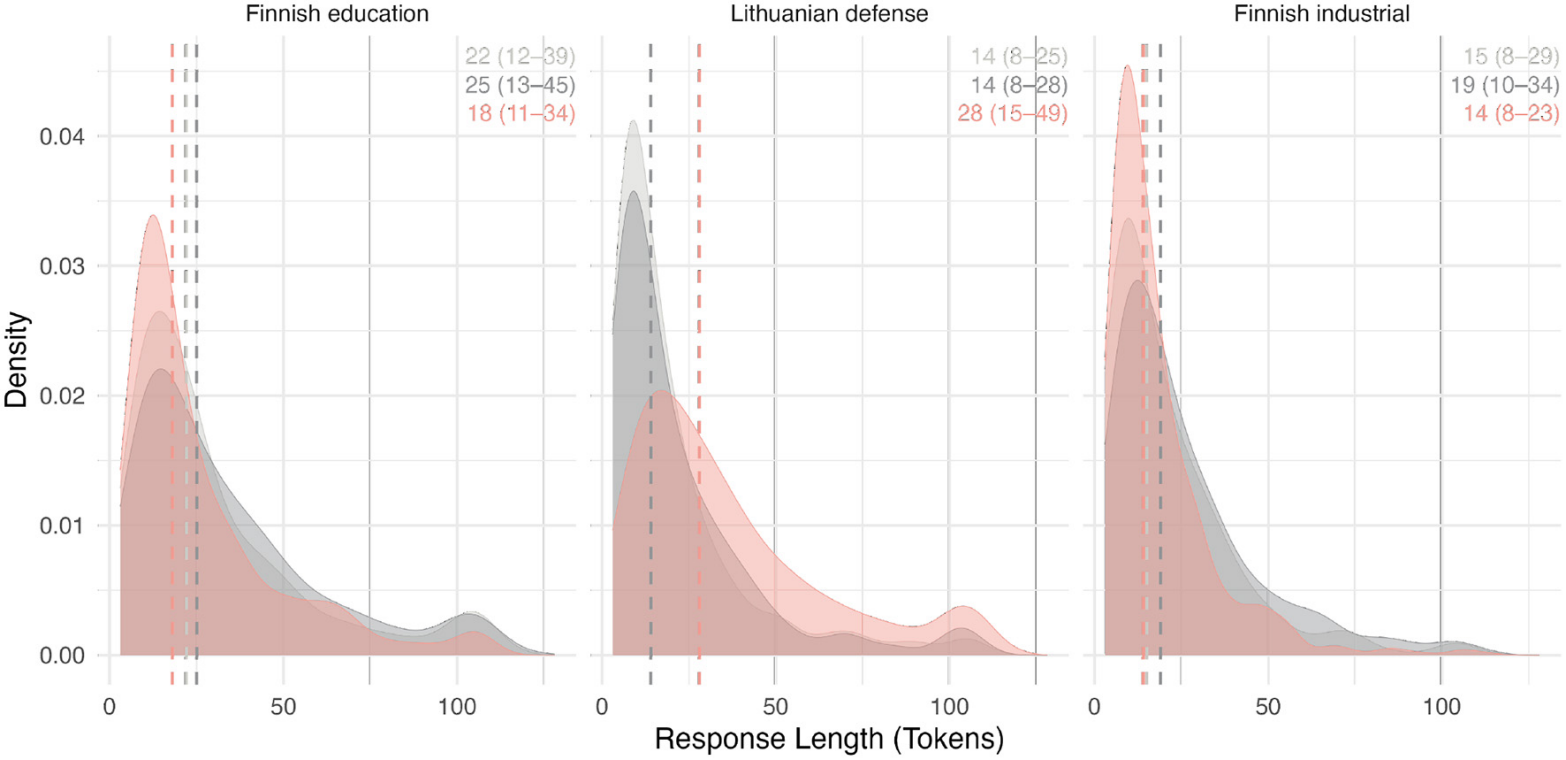
Response-length distributions of 360° open-text comments by dataset and prompt type. Kernel density estimates of response lengths (in tokens) are shown separately for Finnish education (*N* = 4,450), Lithuanian defense (*N* = 3,671), and Finnish industrial sectors (*N* = 2,176). Within each panel, densities are displayed by prompt type (□strengths, □development areas, and □organizational suggestions), with dashed vertical lines indicating median lengths and accompanying text reporting median and interquartile range (IQR). These distributions illustrate the concise nature of 360° narrative feedback and motivate response-level analysis with verbosity normalization.

To characterize the brevity and variability of the text data, the response-length distributions and prompt-wise data amounts were inspected (Figure 2). Median response lengths are short but adequate across datasets, with a long right tail of more elaborated comments. Responses below the preprocessing threshold were excluded as non-informative artifacts. Response lengths were measured in tokens, which are the atomic units processed by our specific data pipeline. The all-MiniLM-L6-v2 model, which is well-suited for text classification tasks involving short, concise text, enables effective analysis of the majority of records in our data.

Because missingness is prompt-dependent (e.g., strengths responses are most frequently completed), analyses are conducted on all available record–prompt observations rather than restricting to complete cases. Additionally, the brevity in the lower quartile of responses implies greater noise at the individual-comment level; accordingly, we emphasize distributional patterns and permutation-based validation rather than interpreting single-response estimates as stable measurements.

The pipeline applies light preprocessing and translation (FI/LT → EN), assigns sentiment with a transformer classifier, and computes construct salience by embedding each response and measuring cosine similarity to seed-phrase representations of Deep Leadership Model (DLM) constructs, followed by normalization for verbosity.

Here, a seed-phrase is a short, theory-derived natural-language statement intended to operationalize a single DLM construct in embedding space, serving as a semantic anchor against which open-text responses are compared. Seed-phrases were drafted from the original DLM construct definitions and DLQ item formulations (Nissinen, 2001), iteratively refined for clarity and coverage, and finally reviewed by domain experts to preserve conceptual fidelity and improve within-construct coverage while avoiding overlap with other constructs. The full seed sets and the seed averaging and exploration procedure are provided in the Supplementary material. Unlike dictionary approaches that rely on enumerating surface forms, the embedding-based approach uses these seed-phrases as semantic anchors, allowing short and lexically variable texts to be positioned relative to theory-defined constructs in embedding space (Garten et al., 2017; Rudkowsky et al., 2018). Thus seed-phrases can be considered as theory-related semantic anchors, rather than entries in a lexical dictionary of DLM constructs.

Technical validation consists of Bayesian regression linking sentiment to overall questionnaire outcomes with controls for prompt type and dataset, convergent validity tests showing higher correlations for matching constructs than non-matching and permutation baselines, and interpretability via role-wise construct profiles.

### 2.1 Validation datasets

Our data comprise Deep Leadership Questionnaire (DLQ) responses and accompanying open-text feedback from leaders who underwent a Deep Lead Ltd. coaching program and their colleagues (giving feedback), between 2007 and 2024. The convenience sample spans leadership development programs in three sectors and countries: Finnish education, Lithuanian defense, and Finnish construction industries.

We analyzed 5,165 DLQ records with at least one open-text response to the prompts on strengths, development, or organization. 360° feedback rater role composition includes self, subordinate, peer, and superior raters. Cohorts were selected to enable contrasts across culturally and organizationally distinct environments. Open-text responses were considered analyzable if they contained at least one full word. Most open-text responses were given in strengths prompt (5,052 responses), whereas the number of records with all open-text responses was much smaller (2,094 responses). Most open-text responses for strengths prompt per role and cohort was given by Lithuanian defense sector subordinates (23.5% of strengths responses). For all open-text responses the most per role and cohort responses were given by Finnish education sector subordinates (23.9% of all responses). Smallest number of response were collected via self-assessement (7.3% of records) where in Finnish education cohort there were only 46 distinct responses.

### 2.2 Measures

The quantitative foundation of this study is the Deep Leadership Questionnaire (DLQ), a psychometrically validated instrument assessing leadership behavior across multiple dimensions within the Deep Leadership Model (DLM) (Nissinen, 2001, 2006).

The DLQ adopts a constructivist perspective, evaluating leadership as a dynamic interaction between individual capability and situational context. It consists of behaviourally anchored factors (Table 1) representing core leadership dimensions such as inspirational motivation, intellectual stimulation, and corrective or passive leadership. The DLQ supports both self-assessment and 360-degree feedback from peers, subordinates, and superiors. Factor scores are summarized into the GLI, a weighted aggregate of theoretical leadership effectiveness.

**Table 1:** Deep Leadership Model constructs, their theoretical definitions, and example seed phrases used for NLP operationalization.

Leadership construct	Theoretical definition	Seed phrase examples
Professional skills (PO)	Demonstrates professional competence, knowledge, and technical leadership skills necessary to lead effectively.	“She has the knowledge and skills of a competent leader.”
Building trust and confidence (BT)	Acts in ways that build mutual trust and respect, often placing group interests above personal interests.	“She will go beyond self-interest for the good of the group.”
Inspirational motivation (IM)	Communicates an appealing vision that inspires and motivates followers to commit to shared goals.	“He is enthusiastic to explain what will happen if we reach our objectives.”
Intellectual stimulation (IS)	Encourages innovation, challenges assumptions, and promotes creativity and critical thinking.	“She is open-minded to new ideas.”
Individualized consideration (IC)	Provides personalized support, coaching, and attention to individual follower needs.	“She gives personal support to subordinates.”
Corrective/controlling leadership (CL)	Monitors performance actively and intervenes early to correct mistakes or deviations.	“He focuses on checking for mistakes.”
Passive leadership (PL)	Avoids decision-making and delays action until problems become critical.	“She is not able to make decisions.”
Satisfaction (SA)	Reflects followers' contentment with the leader's style and behavior.	“I am satisfied with his leadership behavior.”
Effectiveness (EF)	Reflects the leader's ability to achieve desired outcomes and fulfill objectives.	“She meets the training objectives effectively.”
Extra effort (EE)	Ability of the leader to elicit more than expected effort and engagement from followers.	“She is able to make me try harder.”

Construct salience was estimated by computing cosine similarity between open-text response embeddings and seed-phrase embeddings.

The DLQ serves as the backbone of the leadership coaching feedback process and references the NLP-based semantic similarity analysis described in this study. The questionnaire data is supplemented with following open-text prompts:

- *Strengths*: “The strengths of her/his behavior are...”
- *Development*: “Her/his behavior has the following development opportunities...”
- *Organization*: “Cooperation in the organization should be developed as follows...”

### 2.3 NLP analysis workflow

To examine hypotheses, we implemented a reproducible pipeline combining preprocessing, translation, sentiment classification, and embedding-based similarity analysis.

Open-text responses were cleaned using R and Python pipelines. The process removed artifacts, filtered very short entries (fewer than four characters), and corrected formatting errors. The responses in Finnish and Lithuanian were translated into English using GPT-4o via OpenAI's batch API. This translation approach has demonstrated acceptable validity for psychological text analysis in recent studies (Rathje et al., 2024).

All subsequent modeling steps were performed locally in Python 3.12 in a managed environment (Poetry) to ensure reproducibility.

#### 2.3.1 Sentiment analysis and comparison to questionnaire results

Two transformer-based models were used: (i) twitter-roberta-base-sentiment for classifying open-text responses as positive, neutral, or negative; and (ii) all-MiniLM-L6-v2 from SentenceTransformers for computing embeddings of open-texts in each record and construct seed-phrases.

General-purpose models were preferred over fine-tuned ones for better cross-domain robustness. RoBERTa-based sentiment models achieve high accuracy and F1 scores (94%–97%) across varied datasets (Abdal et al., 2023; Kadu et al., 2022), while all-MiniLM-L6-v2 offers optimized efficiency, high-quality embeddings for similarity tasks (Galli et al., 2024).

To test whether open-text response tone correlates with questionnaire-derived leadership index (GLI) we used regression analysis. For that we computed the sentiment score (sentiment) for each record (*i*) that was compared to GLI scores using a Bayesian regression model with a skew-normal likelihood (Equation 1).


$$\begin{array}{l} {\text{GLI}}_{i}\sim {\text{sentiment}}_{ik}\times k\times {\text{dataset}}_{i}, \end{array}$$ (1)

where *k* denotes the question type (Istrengths, Idevelopment, and Iorganization) and *dataset* the cohort. Posterior distributions and 95% credible intervals were estimated in brms (R) (R Core Team, 2025). We treat prompt type as a pre-specified moderator because the prompts differ in evaluative target (leader vs. organization) and are expected to elicit systematically different proportions of evaluative language.

Because GLI ratings in applied 360° feedback are often skewed and with mild ceiling effects, we used a skew-normal likelihood, which extends the Gaussian model by allowing asymmetric residual distributions while retaining an interpretable location parameter on the original GLI scale. This choice was guided by inspection of GLI distributions and posterior predictive checks indicating misfit under a symmetric Gaussian error model.

We estimated the posterior distribution using Markov chain Monte Carlo sampling and treated sentiment as a categorical predictor, fully interacted with prompt type and dataset (Equation 1). Inference is reported via posterior summaries: we compute pairwise contrasts between sentiment categories (negative vs. neutral, negative vs. positive, neutral vs. positive) within each prompt × dataset cell, and report posterior means and 95% credible intervals. Convergence and sampling quality were assessed using standard diagnostics (e.g., $\hat{R}$ and effective sample sizes) and posterior predictive checks.

#### 2.3.2 Semantic similarity analysis and validation

To estimate which DLM constructs each free-text response was associated to, five seed-phrases were designed per DLM construct (see Table 2 for examples). Seed-phrases were derived from the DLM construct definitions and DLQ item formulations, with controlled phrasing variants (e.g., tense and pronoun) to improve representational coverage in embedding space. The full seed lists and documentation of the development process are reported in the Supplementary material.

**Table 2:** Posterior estimates for within-response differences in cosine similarities between “Building trust and confidence” and “Intellectual stimulation” by dataset.

Dataset	Median	95% CI	pd
Education	−0.021	[−0.024, −0.017]	1.000
Defense	0.037	[0.034, 0.040]	1.000
Industrial	−0.013	[−0.019, −0.007]	1.000

Values reflect how strongly each dataset emphasizes BT over IS in open-text responses. Higher positive values indicate greater relative salience of BT. The 95% credible intervals (CI) are shown in brackets. *pd* refers to the probability of direction, i.e., the posterior probability that the effect is positive or negative, whichever is more likely.

Embeddings were computed for each seed-phrase and averaged to form a mean vector per construct, as in Equation 2. The variations in the average vectors were tested using leave-one-out analysis.


$$\begin{array}{l} \mu_{f}^{+}=\frac{1}{P}\underset{p}{\sum }\frac{E\left(s_{fp}^{+}\right)}{∥E\left(s_{fp}^{+}\right)∥} \end{array}$$ (2)

where seed-phrases *p* for each DLM construct *f* go from 1 to *P* (5 in current analysis), *f*: $S_{f}^{+}=\left\{s_{f1}^{+},\ldots ,s_{fP}^{+}\right\}$.

### 2.4 Statistical alignment testing

Embeddings were computed for each open-text response *v*_*ik*_, where *i* is the record and *k* the prompt type. The cosine similarity was calculated between each text embedding and the seed embedding vector $\mu_{f}^{+}$:


$$\begin{array}{ll} s_{ikf} & =v_{ik}^{⊤}\mu_{f}^{+}\in \left[-1,1\right] \end{array}$$ (3a)

$$\begin{array}{ll} z_{ikf} & =\frac{s_{ikf}-{\text{mean}}_{f}\left({\bar{s}}_{ik}\right)}{{\text{sd}}_{f}\left({\bar{s}}_{ik}\right)} \end{array}$$ (3b)

in Equation 3, ${\bar{s}}_{ik}$ is the vector of similarities across constructs within a participant's response, and *z*_*ikf*_ is the normalized salience controlling for verbosity. his controls for the tendency of more verbose responses to show higher similarity to multiple seed phrases.

To assess convergence between semantic salience of the DLM constructs and DLQ factor scores for the constructs we calculated correlations between each pair of similarity scores and questionnaire factor scores across records in each dataset and rater group (3 datasets, 4 rater groups, with 10 matching and 90 non-matching correlations).

Let *Q*_*ig*_ denote the DLQ factor score for construct *g* in record *i*, computed using the standard DLQ scoring procedure. We collect these into the vector $Q_{\cdot g}={\left(Q_{1g},\ldots ,Q_{ng}\right)}^{⊤}$ over the same set of records included in the text analysis. If multiple prompt responses *k* exist within the same record *i*, the associated *Q*_*ig*_ is identical across those prompt responses.

Correlations (Pearson's Ir) were computed by contrasting construct salience scores against matching DLQ factor scores and non-matching salience vs. factor scores:


$$\begin{array}{ll} \rho_{f} & =\rho \left(z_{\cdot f},Q_{\cdot f}\right). \end{array}$$ (4a)

$$\begin{array}{ll} \rho_{fg} & =\rho \left(z_{\cdot f},Q_{\cdot g}\right),f\neq g. \end{array}$$ (4b)

where the operator ρ(·, ·) denotes the correlation coefficient for matching (*f*) and non-matching (*fg*) NLP construct salience and DLQ factor score vectors.

A Gaussian Bayesian linear regression model was used to test whether matching concepts showed higher correlations than non-matching ones. Second, we quantified profile-level alignment at the record level by treating each record as a vector of NLP construct-salience scores and a vector of DLQ factor scores and computing their cosine similarity. To assess whether profile alignment exceeded chance expectations, we constructed a null model by randomly permuting DLQ profiles across constructs while keeping NLP construct-salience profiles fixed, recomputing record-level cosine similarities and the mean cosine similarity under permutation (10,000 iterations).

### 2.5 Interpretable use-cases

To illustrate interpretability (RQ3), two exploratory analyses were conducted on Istrengths responses:

- **Role-wise semantic profiles:** Mean construct salience scores were computed for each rater category (self, subordinate, peer, and superior) to compare how leadership strengths were articulated across rater groups.
- **Salience–questionnaire alignment:** Differences and alignment in NLP construct salience score and DLQ factor scores.

For the role-wise semantic profiles, we computed and compared each construct's NLP construct salience separately for each rater category (sub-ordinates, superiors, self-assessments, and peer evaluations). This enabled the identification of systematic differences in how rater groups articulated leadership strengths relative to leadership dimensions of the theory.

Then, for salience–questionnaire alignment, we examined the relationship between NLP construct salience and DLQ factor scores at the record level. To summarize overall agreement, we computed an *R*^2^ measure between salience scores and DLQ scores across records and constructs. To probe relative differences between constructs, we computed within-record paired contrasts for selected construct pairs (*f*_1_, *f*_2_): Δ*z*_*i*_ = *z*_*i*_*f*__1__−*z*_*i*_*f*__2__ and, analogously, Δ*Q*_*i*_ = *Q*_*i*_*f*__1__−*Q*_*i*_*f*__2__, and then compared these contrast distributions across datasets.

For selected constructs [IBuilding trust and confidence (BT) and IIntellectual stimulation (IS)], we computed within-record paired contrasts. Specifically, Δ*z*_*i*_ = *z*_*i*, BT_ − *z*_*i*, IS_ (NLP construct salience scores) and Δ*Q*_*i*_ = *Q*_*i*, BT_ − *Q*_*i*, IS_ (DLQ scores), and then quantified cohort differences in these contrasts using separate Bayesian Gaussian regression models with dataset-specific means (i.e., no intercept: Δ*z*_*i*_ ~ 0 +dataset and Δ*Q*_*i*_ ~ 0 +dataset). We used weakly informative priors centered at zero and report posterior point estimates with 95% credible intervals and probability of direction (pd) to summarize whether each cohort shows a reliable advantage to one construct in text and/or in DLQ scores.

### 2.6 Algorithm overview

The full analysis pipeline is summarized below. Sentiment and NLP construct salience scores compared with DLQ results were obtained as follows.

**Table:** 

**Inputs**	Open-text responses *v*_*ik*_ for record *i* and question *k* ∈ str, dev, org; DLQ scores *Q*_*if*_; seed sets $S_{f}^{+}={\left\{s_{fp}^{+}\right\}}_{p=1}^{P}$ for constructs *f*.
**Output**	Sentiment label for each *v*_*ik*_; construct salience *z*_*ikf*_; validation statistics.

1. **Clean and filter:** Remove artifacts; drop responses with length ≤ 4.
2. **Translate:** Language ∈{FI, LT}, translate to EN via batch GPT-4o.
3. **Sentiment:** Classify with twitter-roberta-base-sentiment to get label ∈ {neg, neu, pos} and score.
4. **Embed text:** Encode using all-MiniLM-L6-v2.
5. **Embed seeds:** Encode embeddings per construct, using concatenated seed-phrases.
6. **Construct vector:** Average seeds to obtain centroid.
7. **Raw salience:** Compute cosine similarity $s_{ikf}=E{\left(v_{ik}\right)}^{⊤}\mu_{f}^{+}\in \left[-1,1\right]$.
8. **Normalize:** Calculate $z_{ikf}=\frac{s_{ikf}-{\text{mean}}_{f}\left({\bar{s}}_{ik}\right)}{{\text{sd}}_{f}\left({\bar{s}}_{ik}\right)}$ to control verbosity.

Then hypotheses were tested as follows.

1. **RQ1 (Sentiment**→**GLI):** Fit Bayesian skew-normal model (GLI_*i*_ ~ sentiment_*ik*_ × *k* × dataset_*i*_) and report contrasts and 95% CrIs.
2. **RQ2 (Convergent validity):** Compute matched vs non-matched correlations. Fit Bayesian contrast (matched > non-matched); Permute *Q* (*N* = 10,000) to form null alignment; report observed vs null gap.
3. **RQ3 (Interpretability):** Aggregate by role and dataset; visualize construct profiles; Compute variation explained in DLQ scores by NLP construct salience scores across records. Examine within-record differences for selected constructs (i.e., BT vs. IS) for both analysis methods.

## 3 Results

Having introduced the data, measures, and pipeline, the results evaluate the method on three criteria that translate hypotheses into measurable targets. RQ1 assesses whether sentiment from open-text relates to overall leadership quality (GLI); RQ2 tests convergent validity by comparing construct salience from NLP pipeline to DLQ factor scores against non-matching and permutation baselines; RQ3 demonstrates interpretability of alignment and misalignment between NLP construct saliences and DLQ factor scores.

### 3.1 Seed-phrase embedding diagnostics

Before presenting the main analyses results, we examined the internal stability and discriminability of the seed-phrase anchors used to represent the DLM constructs. Because construct salience is computed by comparing text embeddings to mean seed-phrase embeddings, it is important that these anchors are not overly sensitive to individual seed-phrases and that different constructs remain distinguishable in embedding space.

To assess robustness, we conducted a leave-one-out (LOO) stability analysis. For each construct, the mean seed embedding was recomputed after removing each seed-phrase in turn, and the cosine similarity between the full anchor and the leave-one-out anchor was measured. Stability was summarized as the directional change 1 − cos(μ_*c*_, μ_*c*, −*i*_). Across constructs, the mean LOO deviation was small (mean = 0.022, SD = 0.008), indicating that the construct anchors were highly stable and not dominated by any single seed-phrase.

To examine potential construct leakage (i.e., insufficient separation between construct anchors), we analyzed cosine similarities between construct anchors. The mean off-diagonal cosine similarity between different constructs was 0.523 (maximum = 0.762), reflecting the expected semantic relatedness among leadership constructs. However, discriminability remained substantial: the average separation between each construct and its most similar alternative (margin $=1-\max_{d\neq c}\cos\left(\mu_{c},\mu_{d}\right)$) was 0.327 (SD = 0.080). Because no external benchmark exists for expected overlap among DLM constructs, these values should be interpreted descriptively rather than against a fixed threshold. But together, these diagnostics indicate that while the constructs occupy a related semantic region in embedding space, the seed-based anchors remain sufficiently distinct to support construct-level salience estimation.

All cosine similarities reported in these diagnostics are computed using the all-MiniLM-L6-v2. Accordingly, the robustness and discriminability statistics are directly comparable to each other. At the same time, these values should be interpreted as model-conditional diagnostics—absolute cosine levels may differ under alternative embedding models, even though the underlying procedures remain identical.

### 3.2 Sentiment analysis (RQ1)

The relationship between sentiment label and the General Leadership Index (GLI) confirms that textual tone in open-text reflects questionnaire-based leadership ratings. As expected, negative sentiment in the Istrengths question was associated with lower GLI scores. Other prompts showed weaker or no associations, except in the Lithuanian dataset, characterized by a more hierarchical context, where more negative organizational views also coincided with lower GLI values.

**Figure 3:**
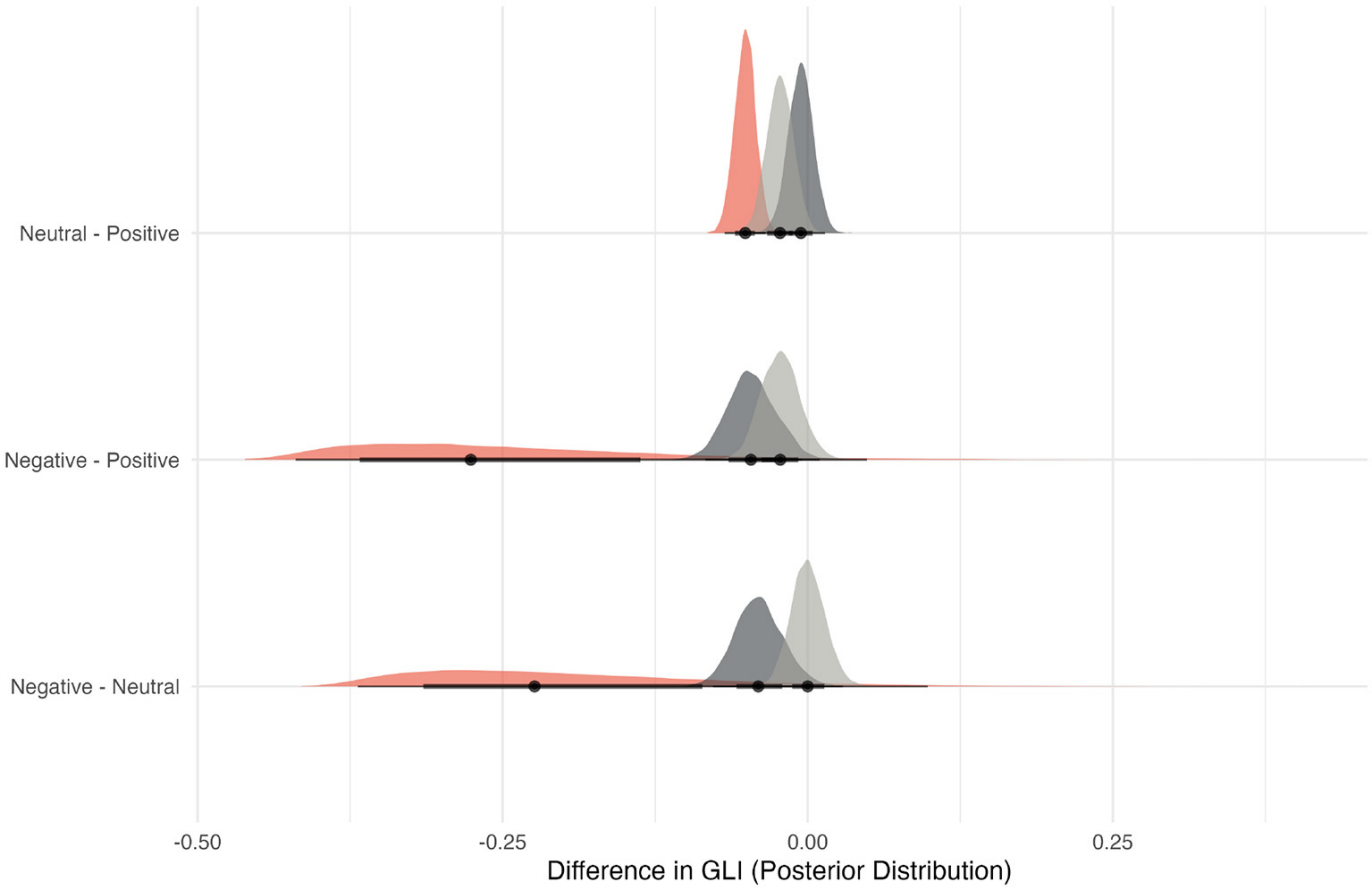
Posterior distributions of differences in General Leadership Index (GLI) scores between each sentiment label in the data for each open-text question type (□strengths, □development areas, and □organizational suggestions). This contrast highlights how negative sentiment in “The strengths of her/his behavior are” responses corresponds to lower GLI scores, supporting the construct validity of interpretation of sentiment analysis. The black bars on X-axis represent 95% credible intervals.

A Bayesian regression model with a skew-normal likelihood was fitted with GLI as a function of sentiment, prompt type, and dataset. As shown in Figure 3, negative Istrengths responses corresponded to a mean GLI decrease of 0.23–0.26 points relative to neutral or positive responses (95% credible interval excluded zero) for all data. Sentiment effects for Idevelopment areas and Iorganization questions were smaller and uncertain, with intervals often overlapping zero, consistent with these prompts being less purely evaluative than strengths descriptions.

As a supplemental consistency test across cohorts, we fitted a simple regression model to our data and examined the effect of the dataset factor on GLI. While the large sample size resulted in a statistically significant effect (*p* < 0.001), the practical impact was negligible, with the dataset factor accounting for only 3% of the partial variance ($\eta_{p}^{2}=0.03$). This suggests that the results are highly consistent across the cohorts.

### 3.3 Semantic similarity analysis (RQ2)

**Figure 4:**
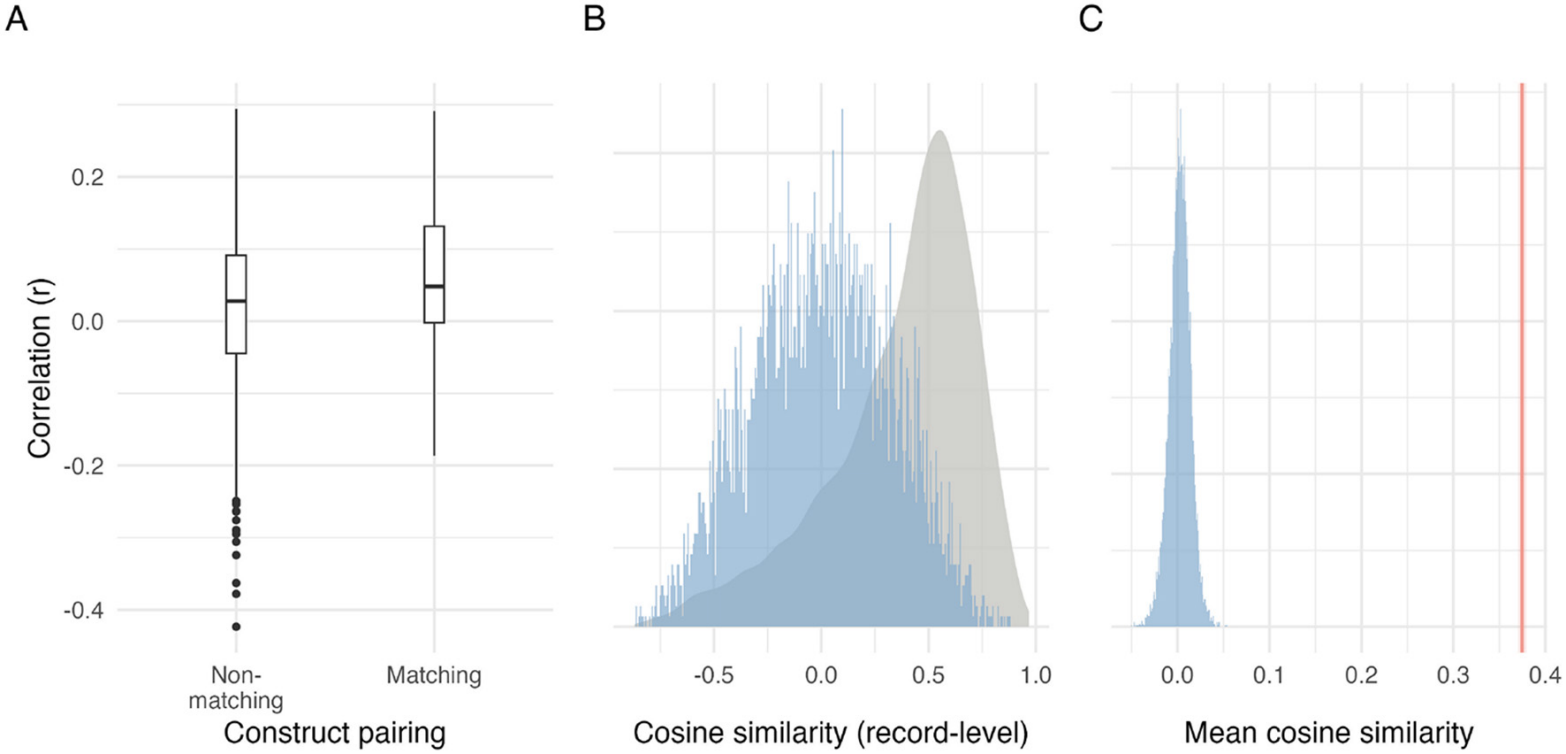
Semantic alignment between text-derived construct salience and DLQ factors: construct-wise correlations and profile-level permutation baselines. **(A)** Distributions of raw construct-wise correlations across record–prompt observations: for each DLQ factor, we correlate its score with the corresponding NLP construct-salience score (theoretically matching) and with non-corresponding salience scores (theoretically non-matching). This panel summarizes the raw pairwise construct–construct associations. **(B)** Record-level profile alignment (heterogeneity across records): the □density shows the distribution of cosine similarities between each record's full text-derived construct-salience vector and its DLQ factor-score vector; □histogram illustrates one shuffled null draw obtained by randomly permuting DLQ profiles across constructs (NLP profiles fixed). **(C)** Permutation test on mean profile alignment: □line denotes the observed mean of record-level cosine similarities (with SE), and □histogram shows the null distribution of mean alignment scores from 10,000 permutations, indicating that observed profile alignment exceeds what is expected under random pairing. Cosine similarity in **(B, C)** quantifies alignment of construct profiles of a record and is distinct from the text–seed cosine similarity used earlier to derive construct salience.

To evaluate how effectively embeddings of open-text responses capture leadership constructs detected by the DLQ, cosine similarities between embedding vectors of open-text responses and construct seed vectors were correlated with DLQ factor scores. Matched correlations—where both measures reflected the same construct—were consistently positive and higher than non-matched pairs, supporting meaningful semantic alignment (Figure 4A).

A Bayesian regression estimated the average matched–non-matched difference as +0.03 (95% CrI [0.01, 0.05], *pd* = 99.63%). Thus, NLP construct salience reliably aligned with corresponding DLQ scores, although the magnitude was modest.

To formally benchmark profile-level alignment against chance, we performed a permutation test by shuffling DLQ profiles across records (10,000 iterations) and recomputing the mean record-level cosine similarity. The observed mean alignment lies far in the right tail of the permuted mean distribution (Figure 4C), indicating that the average semantic alignment between NLP-derived salience profiles and DLQ profiles is unlikely to arise under random pairing. Importantly, the record-level density in Figure 4B visualizes the heterogeneity across records: while some records show weak or even negative alignment, the distribution as a whole is shifted upward relative to the shuffled null distribution, supporting the interpretation that many individual record–prompt responses carry detectable theory-consistent signal despite substantial record-to-record variability.

As supplementary significance checks for both Figures 4B, C, we compared observed cosine-similarity patterns against shuffled DLQ baselines. For the record-level distributions in Figure 4B, observed cosine similarities were stochastically larger than those under shuffled pairing (*p* < 0.001, Wilcoxon rank-sum), given the sample size this is unsurprising. We treat the permutation test on the mean alignment in Figure 4C as the primary evidential benchmark, and the record-level test as a supplementary indication that the observed similarities are generally higher than chance.

### 3.4 Practical use cases (RQ3)

**Figure 5:**
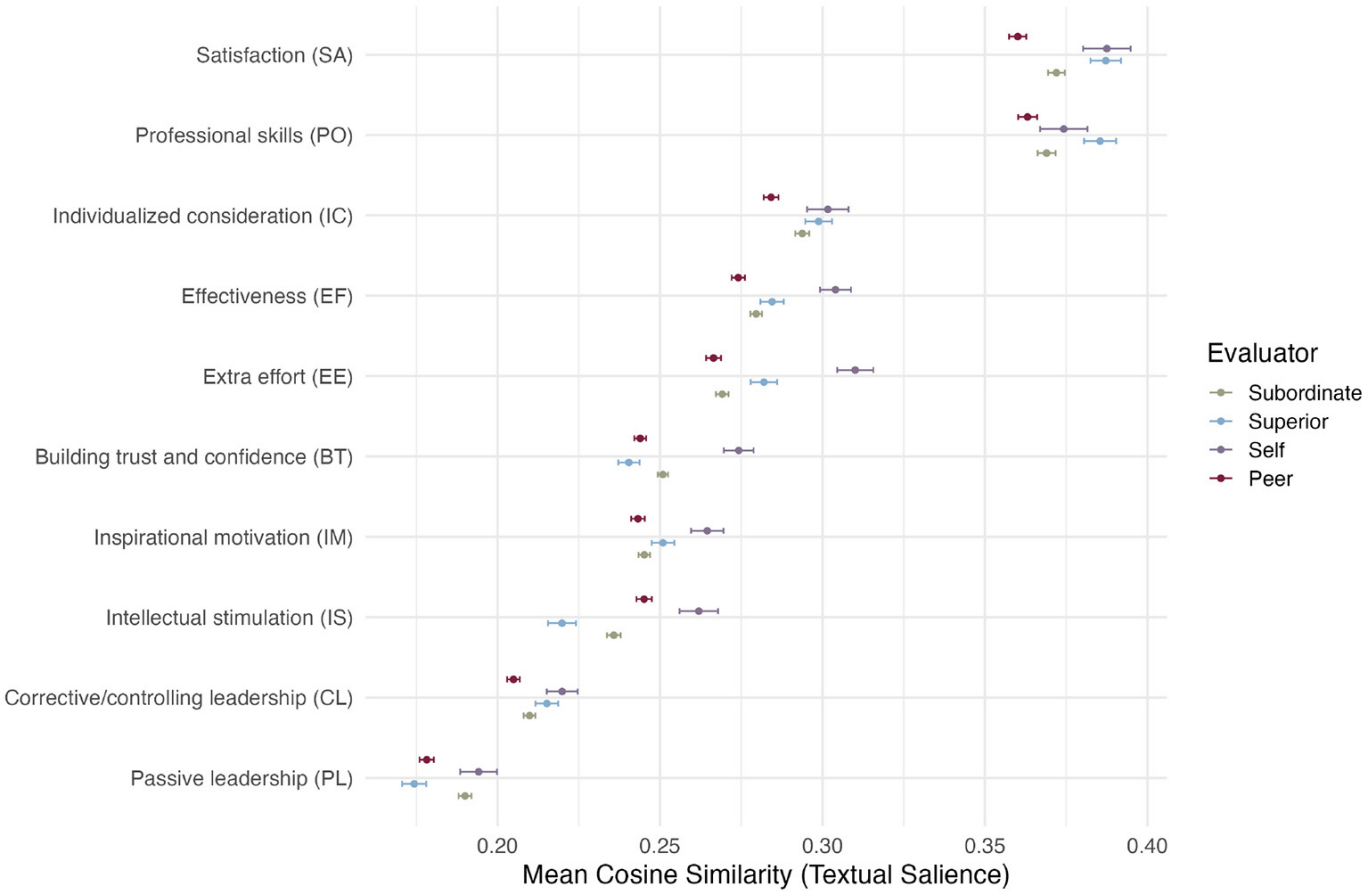
Textual salience (NLP construct salience score, see Equation 3b) (•) for each leadership constructs by rater group in the Istrengths prompt responses. The salience value indicates how much each leadership construct from the DLM is expressed in the open-text. Bars show standard errors across responses within rater group. Colors denote the rater group as follows; •for subordinate responses, •for superiors, •for self evaluations, and •for feedback from peers.

To examine RQ3, we illustrate interpretability and practical applications of the results. Figure 5 illustrates differences in Istrengths responses across rater roles (self, subordinate, peer, and superior). For instance, coacheés emphasized constructs such as IIntellectual stimulation more than evaluators that were not part of the coaching program, suggesting subtle role-based differences in how leadership behaviors are described.

Beyond overall convergence (RQ2), comparing text-derived construct salience with DLQ factor scores enables a distinction between constructs that are explicitly expressed in narrative feedback and those that are endorsed in ratings but remain comparatively unspoken in text. We operationalized this by examining the overall NLP–DLQ agreement (*R*^2^ = 0.11 across records/constructs), and within-record paired contrasts between focal constructs (here, IBuilding trust and confidence (BT) vs. IIntellectual stimulation (IS)), computed in parallel for NLP construct salience scores (Δ*z*) and DLQ scores (Δ*Q*). This contrast-based view focuses on relative emphasis within the same record, making it robust to differences in scale units between cosine similarity and questionnaire scores.

In the Lithuanian defense sector, open-text salience for BT and IS tracked their DLQ scores. By contrast, in the Finnish cohorts, IS was more pronounced in open-text salience, whereas BT dominated DLQ scores (Tables 2, 3).

**Table 3:** Posterior estimates for within-response differences in DLQ factor scores between “Building trust and confidence” and “Intellectual stimulation” across datasets.

Dataset	Median	95% CI	pd
Education	0.119	[0.094, 0.144]	1.000
Defense	0.069	[0.048, 0.090]	1.000
Industrial	0.161	[0.122, 0.200]	1.000

Values reflect the average advantage of DLQ scores (BT over IS) within each dataset, with 95% credible intervals (CI) in brackets and pd is the probability of direction.

Although these differences are small in magnitude (≈3% on average for both DLQ scores and cosine similarities), their consistent direction and statistically rigorous evidence support meaningful within-response contrasts.

## 4 Discussion

This study demonstrates the feasibility and added value of integrating modern NLP techniques, specifically sentiment analysis and semantic embeddings, with validated psychometric instruments. Our findings show that this approach complements structured leadership assessments such as the Deep Leadership Questionnaire (DLQ), while also revealing interpretive nuances from open-ended feedback.

Sentiment analysis results indicate that the emotional tone of open-text leadership evaluations aligns meaningfully with overall leadership ratings (GLI), particularly in responses to the Istrengths question. Previous research has shown that NLP-based analysis of open-text responses can outperform structured assessment approaches in extracting sentiment (Sikström et al., 2024). We build on this by demonstrating that even when not explicitly prompted the open-text can be used to assess emotional aspects in target constructs. This supports the idea that sentiment classifiers can serve as lightweight proxies for leadership quality in feedback-rich but resource-limited evaluation settings.

In contrast, sentiment expressed in Idevelopment areas and Iorganizational improvement suggestions showed weaker or inconsistent links with GLI. We interpret this pattern as a consequence of prompt framing. The development-areas question invites constructive, forward-looking critique irrespective of overall leadership quality, and it can be expressed in either negatively valenced language (problem-focused) or positively valenced language (solution- or growth-focused), reducing the specificity of sentiment as an index of leadership endorsement. The organizational improvements prompt further shifts the target of evaluation from the individual leader to systemic constraints and processes, which are often discussed in a more neutral, problem-solving register. Thus, in these prompts sentiment becomes comparatively orthogonal to overall leadership quality, even though the responses may still carry informative semantic signal about what respondents consider important.

These distinctions highlight the need for context-aware interpretation of sentiment within structured feedback instruments. From a coaching perspective, these patterns can guide more targeted interventions. Divergences between positive open-text feedback and low DLQ scores, for example, may signal cultural or contextual emphasis that the standard model under-represents. Identifying such mismatches can support more personalized, context-sensitive development planning than questionnaire results alone.

It has been shown that NLP methods assessing semantic similarities can validate internal structures of leadership models (Arnulf et al., 2014). Our results extend the use of modern NLP analysis in the free-text discussions. Specifically, our semantic similarity analyses further show that NLP methods can reliably detect leadership constructs in narrative feedback. Although the correlations between text-derived construct salience and DLQ factor scores were modest, their directionality and consistency were statistically robust, supporting the convergent validity of NLP-based construct estimation.

Finally, NLP-derived measures revealed additional patterns beyond questionnaire scoring. At both individual and group levels, participants expressed the constructs in ways that were not fully reflected in their DLQ profiles.

First, the rater group differences in Figure 5 provide a useful sanity check for interpreting NLP construct salience scores. The groups plausibly differ in their exposure to the Deep Leadership Model (DLM): coachees (and in many programs also their superiors) are explicitly introduced to the DLM constructs during training, whereas other colleagues may have less direct exposure. Consistent with this, self-evaluation narratives show higher salience for DLM constructs, suggesting that greater exposure is associated with a more DLM-congruent framing of open-text feedback. In other words, the result captures differences in what respondents choose to emphasize in their narratives, rather than providing a direct behavioral measure.

Second, DLQ factor scores capture endorsed strength levels (how strongly a rater agrees that a behavior is present), whereas narrative salience reflects communicative emphasis (which behaviors the rater chooses to mention when summarizing strengths). The two need not coincide: some constructs may be rated highly yet remain relatively implicit in text because they are taken-for-granted or normatively expected, while other constructs may be voiced disproportionately because they are especially salient, distinguishing, or development-relevant in that context. In our data, this distinction is visible in the BT–IS contrasts: in the Lithuanian defense cohort, BT's relative advantage over IS was consistent in both DLQ scores and open-text salience (alignment), whereas in the Finnish cohorts BT dominated DLQ scores but IS was more pronounced in open-text (systematic mismatch). Since both scores (DLQ and NLP) are expressed in arbitrary units (Likert and normalized cosine similarity, respectively), we emphasize the direction and consistency of within-record contrasts rather than absolute magnitudes.

These discrepancies suggest that open-text responses surface locally or culturally salient leadership themes that structured items may under-represent. While DLQ constructs are theoretically grounded and psychometrically validated, they reflect an externalized model of leadership. By contrast, open narratives reveal how leadership is perceived and enacted within specific cultural and organizational contexts.

In practice, NLP analysis can therefore enhance the contextual responsiveness of leadership development. By combining structured scores with text-based insights, coaches and trainers can identify both measurable competencies and emergent themes meaningful within a particular setting.

Methodologically, our results advocate for a mixed-mode analytical paradigm that joins psychometric rigor with NLP-enabled flexibility. The pipeline supports both validation of existing constructs and inductive detection of unmodeled dimensions. Beyond leadership research, the same framework can be applied to educational assessment, psychological evaluation, or any domain pairing validated instruments with open-text responses. Its modular, reproducible design also facilitates cross-sector and multilingual adaptation. We used general-purpose transformer models to avoid relying on domain-specific tuning. The pipeline is modular and can incorporate alternative or fine-tuned models; however, model changes should be accompanied by explicit version reporting and re-validation against the questionnaire anchors to preserve comparability over time.

In our framework, the NLP construct salience score quantifies what raters choose to emphasize in free-text responses, while DLQ scores measure the rated intensity of behaviors. Because these processes differ, and considering the noise inherent, especially, in short or incomplete open-text responses, we emphasize the importance of examining distributional patterns across records rather than drawing conclusions from any single response. Additionally, given the exploratory nature of seed-phrase development, we caution against treating these individual salience estimates as stable or definitive measures for any given leader. Instead, these estimates serve as complementary diagnostic information, which should be considered alongside the DLQ's more structured, ratings-based assessment.

Several limitations warrant consideration. We cannot model leader-level nesting (multiple raters per leader) because specific coacheé identification is unavailable; standard errors may therefore be optimistic if substantial within-leader dependence exists. The next boundary condition concerns discursive context. Our workflow is suitable for short responses in the sense that it represents each comment as a standalone embedding and derives construct- or topic-level summaries by aggregating across many such units. This design supports scalable analysis of brief 360° comments, but it does not, without modification, model discourse-level phenomena such as conversational dependencies. The present dataset therefore serves primarily as a proof-of-concept for validating theory-anchored text metrics under realistic organizational feedback conditions.

Machine translation may introduce subtle semantic distortions, making cross-language comparisons tentative. Cosine similarity, in turn, reflects semantic proximity rather than exact semantic equivalence and condensing DLQ constructs into a handful of seed-phrases may oversimplify their conceptual richness.

Furthermore, it assumes linguistic consistency and conceptual clarity that may not universally apply. Transformational and transactional concepts, while established in leadership theory, vary subtly by context, language, and organizational culture, potentially complicating their direct semantic capture. Our methodology thus provides a theoretically sound yet contextually flexible approach, while being constrained to the semantic representations of the underlying models. Cultural differences observed in construct salience (e.g., heightened emphasis on Intellectual Stimulation in Finnish contexts) are presented as illustrative rather than definitive. From an emic–etic perspective, such patterns may reflect locally salient interpretations of leadership that standardized instruments only partially capture, rather than stable cross-cultural differences in underlying constructs.

Another limitation is that our open-text prompts were not originally designed as semantic questions optimized for construct measurement. Prior work suggests that prompt design can materially affect the validity of language-based measures (Kjell et al., 2019). Future work could compare alternative prompt formats (e.g., semantic questions targeting each DLM construct) against the existing DLQ items, and test whether language-based measures outperform, match, or complement rating scales under controlled conditions. Furthermore, incremental validity studies are necessary to bridge the explanatory gap between NLP-derived metrics and DLQ results. Such studies should examine predictive validity and practitioner-oriented indicators, such as rank-order agreement between NLP-derived and questionnaire-based construct profiles, and potentially classification accuracy for dominant constructs.

Future work should explore how AI systems can actively assist feedback interpretation, e.g., by highlighting actionable insights or clustering themes across participants. Such AI-supported enhancements could make feedback more specific, actionable, and developmentally targeted, enriching current assessment practices in leadership coaching and beyond.

## 5 Conclusion

This study shows that modern NLP techniques—specifically sentiment analysis and semantic similarity analysis using transformer-based methods—can enrich leadership assessment by extracting meaningful patterns from open-text responses. These methods uncover latent leadership dimensions that may be overlooked or under-represented in structured questionnaire data, thereby offering a complementary lens on behavior and perception.

While our illustrative findings, such as the heightened salience of IIntellectual stimulation in Finnish contexts, demonstrate interpretive potential, the central contribution lies in the methodology. The proposed pipeline is modular, language-agnostic, and adaptable across leadership frameworks or any validated psychometric instrument accompanied by open-text data. By testing semantic alignment between narratives and psychometric constructs, we offer a replicable framework for integrating qualitative and quantitative evidence in psychometric research.

In sum, the method expands the analytic toolkit for studying meaning in leadership and beyond. It enables psychometric constructs that are traditionally confined to numeric validation to be examined through language itself, moving from asking to listening while still incorporating rigorous psychometric measures for analysis.

## Data Availability

The datasets presented in this article can be made available upon request. Requests to access these datasets should be directed to lauri.v.ahonen@helsinki.fi.
